# Distorted Self-Perceived Weight Status and Underestimation of Weight Status in Diabetes Mellitus Type 2 Patients

**DOI:** 10.1371/journal.pone.0095165

**Published:** 2014-04-15

**Authors:** Victor Mogre, Robert Abedandi, Zenabankara S. Salifu

**Affiliations:** 1 Department of Human Biology, School of Medicine and Health Sciences, University for Development Studies, Tamale, Ghana; 2 Department of Allied Health Sciences, School of Medicine and Health Sciences, University for Development Studies, Tamale, Ghana; McMaster University, Canada

## Abstract

**Objectives:**

Diabetes mellitus type 2 (DM 2) patients' self-perception of their weight status is very critical in diabetes care. We sought to investigate perception of weight status in a sample of 200 DM 2 patients attending an outpatient clinic at a Teaching Hospital and compared it with their *BMI-measured weight status*, with a focus on underestimation of their weight status. Factors associated with underestimation of weight status in this sample were also explored.

**Methods:**

Using a cross-sectional design, anthropometric and clinical variables were assessed using appropriate tools. Questionnaires were used to collect socio-demographic data and self-perception of weight status. Self-perceived weight status was compared to *BMI-measured* weight status by cross-tabulation, Kappa statistics of agreement and χ2 for trend analysis. Both univariate and multiple logistic regression analysis were conducted to identify factors associated with underestimation of weight status.

**Results:**

The prevalence of general *overweight/obesity* and abdominal obesity was 32.0% (n = 64) and 58.0% (n = 116) respectively. Generally, 58.0% (n = 116) of the participants had a distorted weight perceived weight status in which 77.6% (n = 90) underestimated their weight status. Factors associated with underestimation of weight status were being *overweight/obese* (AOR = 22.9, 95% CI = 8.30–63.07, p<0.001), not married (AOR = 3.7, 95% CI = 1.50–9.17, p = 0.005) and never tried to lose weight (AOR = 6.9, 95% CI = 2.35–19.97, p<0.001). Participants aged over 40 years and those being hyperglycaemic were not significantly associated to underestimation of weight status.

**Conclusion:**

We found a substantial discordance between *BMI-measured* and self-perceived weight status. Factors that were associated with underestimation of weight status were being; *overweight/obese*, not married and never tried to lose weight. Diabetes patients should be provided with information about weight guidelines.

## Introduction

Globally, diabetes mellitus type 2 (DM 2) is increasing in epidemic proportions and has become an important cause of mortality [1]. With about 6% of Ghanaian adults affected with DM 2 [2], it has been shown to be the most commonest form of diabetes in sub-Saharan Africa [3], [4] constituting 90–95% [5] of all diabetes cases.

Overweight/obesity is the commonest and major potentially modifiable risk factor among adults with DM 2 [6], [7]. Epidemiological studies have established that DM 2 patients have a higher body weight than control populations indicating a relationship between obesity and the development of diabetes [8]. An overweight/obesity prevalence ranging between 50–90% have been reported among DM 2 patients in Ghana [2], Nigeria [9], India [10] and the US [11].

Weight loss and maintenance are critical in diabetes care. Modest weight loss leads to considerable improvements in all aspects of cardiovascular disease risk and other related conditions [12]. Critical to the adoption of weight management recommendations is the motivation to lose weight. The motivation to lose weight is the recognition by overweight/obese individuals that they are overweight/obese and therefore are at risk for serious negative health consequences [13]. This recognition is influenced by the individual's perception of his/her weight, which could be distorted. A distorted weight perception occurs when one self-perceives his/her weight status to be found in a different category (normal weight, overweight, obese) than would have been if determined by an objective method of measurement [14], [15].

Underestimation of weight status, a form of self-distorted weight status has been shown to contribute to denial or minimization of current weight being a health risk [16], [17], [18]. This may result in the difficulty of promoting weight loss [19] or inattention to obesity management in diabetes care [20] which can result in worse disease and clinical outcomes. Self-management is a critical component of diabetes care [6] and patients' understanding of weight-related health risk may be a critical step toward setting healthy lifestyle goals and effective weight management [11]. It is therefore pertinent to make DM 2 patients aware of their weight status and understand the importance of adopting self-care behaviors to reduce weight and prevent complications [21]. Distorted self-perceived weight status can have an influence on the clinical outcomes (such as blood pressure, fasting plasma glucose among others) of diabetes patients.

Factors such as age, sex, socio-economic status, marital status, and weight loss attempts have been shown to influence distorted underestimation of weight status in the general population [14], [15], [22], [23]. In Ghana and other African countries distorted self-perceived weight status have been associated with cultural and social norms [15], [24]. However, studies examining distorted self-perceived weight status in DM 2 patients and associated factors are sparse and unavailable in developing countries like Ghana. In addition, studies on the effect of distorted weight status on clinical parameters (like blood pressure and fasting plasma glucose) are limited.

We therefore sought to investigate perception of weight status in a sample of 200 DM 2 patients receiving care from an outpatient clinic at a Teaching Hospital and compared it with their *BMI-measured weight status*, focusing on the underestimation of weight status. Furthermore we explored factors associated with underestimation of weight status in this sample of DM 2 patients.

## Materials and Methods

### Ethics statement

All data collection methods complied with the guidelines of the Ethics Committee of the University for Development Studies, School of Medicine and Health Sciences, Ghana, which subsequently approved the study. Each participant gave verbal informed consent prior to participation, since most of them could neither speak nor read the English language. Informed consent was recorded by ticking on a questionnaire indicating that “informed consent granted” or “informed consent not granted”. All informed consent procedures were approved by the Ethics Committee of the University for Development Studies, School of Medicine and Health Sciences, Ghana.

### Participants

This cross-sectional study was conducted from April – June 2013 at an outpatient diabetes clinic of the Tamale Teaching Hospital in Tamale, Ghana. All previously diagnosed diabetes mellitus type 2 patients that sought for care from the Hospital's diabetes clinic, during the study period were eligible to participate in the study. Two hundred and fifteen participants were approached; 200 of them consented to the study, yielding a response rate of 97.6%.

### Anthropometric parameters

Anthropometric parameters such as weight, height, waist and hip circumferences were taken. The weights of subjects were measured to the nearest 0.1 kg using the UNICEF electronic scale manufactured by seca. All scales used were calibrated with a standard weight prior to use. The heights of participants, without shoes on, were measured using a wall-mounted microtoise calibrated to the nearest 1 cm. Body mass index (BMI) was calculated as: BMI (kg/m^2^) =  weight (kg)/[height (m^2^)]. Overweight and obesity were defined using the current World Health Organisation definitions: underweight: BMI<18.5 kg/m^2^, normal weight: BMI 18.5–24.9 kg/m^2^, overweight (pre-obese): BMI 25–29.9 kg/m^2^ and Obese: BMI>30 kg/m^2^ [25]. Waist circumference (WC) was measured midway between the inferior angle of the ribs and the suprailiac crest [26] to the nearest 1 cm using a non-stretchable fibre-glass measuring tape (Butterfly, China). During the measurement, participants stood in an upright position, with arms relaxed at the side, feet evenly spread apart and body weight evenly distributed in accordance with the WHO expert consultation report on waist circumference and waist-to-hip ratio [26]. Abdominal obesity was determined as a waist circumference >102 cm in men and >88 cm in women according to the World Health Organization cut-off points and risk of metabolic complications for Waist circumference [26].

### Clinical parameters

Clinical variables such as systolic blood pressure (SBP), diastolic blood pressure (DBP) and Fasting Plasma Glucose (FPG) were recorded from the personal health record files of the diabetic patients. Normoglycaemia was defined as a fasting venous plasma glucose concentration of less than 6.1 mmol^−1^ [27]. Hyperglycaemia was classified as FPG≥6.1 mmol^−1^ [27]. Similarly, hypertension denoted a mean BP≥140/90 mmHg and/or documented anti-hypertensive treatment [28].

Parameters such as gender and duration of diabetes were also obtained from the patients.

### Self-Perception of weight status

Information on self-perceived weight status was obtained by means of a previously validated self-administered questionnaire [15]. Weight status perception was assessed using the question: “Do you think your weight is: about the right weight, underweight, overweight or obese”. Socio-demographic data such as gender, age, marital and educational status as well as weight management behaviours were also obtained using the questionnaire.

### Distorted perception of weight status

As an initial step, distorted perception of weight status was measured by determining the accuracy of participants' self-perceived weight status compared to their *BMI-measured* weight status. Distorted self-perceived weight status was identified when the self-perceived weight status category (about the right weight, underweight, overweight or obese) did not correspond to the *BMI-measured weight status* category (underweight, normal weight, overweight/obese) [14], [15]. Furthermore, two forms of distorted self-perceived weight status were identified (overestimation and underestimation of self-perceived weight status). Underestimation of weight status was determined when the self-perceived weight status category was found to be lower than the *BMI-measured weight* status category. The reverse held true for overestimation of weight status.

### Statistical analysis

All statistical analysis were conducted using GraphPad Prism version 5 (GraphPad software, San DiegoCalifornia USA, www.graphpad.com) and two-tailed *p* values. Descriptive statistics (means ±SD and proportions) were used to characterize the anthropometric, clinical and socio-demographic parameters. Self-perceived weight status was compared to the *BMI-measured* weight *status* by cross-tabulation, Kappa statistics of agreement and χ2 for trend analysis. Using simple logistic regression, we conducted univariate analysis to assess the effect of socio-demographic data (gender, age, marital status and educational status), weight management behaviors (ever tried to lose weight and ever tried to gain weight), anthropometric (abdominal obesity and general overweight/obesity (BMI)) and clinical parameters (hypertension status and Blood glucose status) on underestimation of weight status (dependent variable) and the results reported as crude odds ratio at 95% confidence interval (CI). Significant independent variables (aged over 40 years, married, ever tried to lose weight, hyperglycaemic and general overweight/obese (BMI)) from the univariate model were entered into a multivariate model and analyzed using multiple logistic regression and results reported as adjusted odds ratios (AOR) at 95% CI.

## Results

### Demographic, anthropometric and clinical parameters

Contained in table 1 are the descriptive statistics of the demographic, anthropometric and clinical parameters of the 200 participants. Eleven percent of the study participants were less than 40 years of age; less than 15% of the study participants were unmarried; and less than 40% had attained a high level of education. Mean ±SD BMI values corresponded to 24.28±4.64 Kg/m^2^ in men and 23.74±4.66 kg/m^2^ in women. Women had higher mean waist circumference than men. Generally, participants had mean ±SD values greater than 120 mmHg for systolic pressure; 80 mmHg for diastolic pressure; 6 m/mol for fasting plasma glucose; and 4 years for duration of diabetes.

**Table 1:** Mean ±SD of anthropometric and clinical parameters of the participants.

Variable	Total (n = 200)	Men (n = 46)	Women (n = 154)	P value
Age (years)	56.2±12.13	52.83±10.89	57.22±12.33	0.031
Age (over 40 years)	178 (89.0%)	38 (82.6%)	140 (90.9%)	0.176
Married	152 (76.0%)	34 (73.9%)	118 (76.6%)	0.007
Low educational level	132 (66.0%)	26 (56.5%)	106 (68.8%)	0.156
BMI (Kg/m^2^)	23.86±4.64	24.28±4.56	23.74±4.66	0.716
WC (cm)	95.99±15.63	85.17±13.98	99.22±14.64	<0.001
Systolic BP (mmHg)	122.80±16.17	127±12.09	121.6±17.04	0.014
Diastolic BP (mmHg)	84.50±13.85	88.26±11.80	83.38±14.24	0.023
FPG (m/mol)	7.94±2.81	8.07±2.77	7.90±2.83	0.904
DD (years)	5.23±5.00	6.37±4.96	4.88±4.98	0.023

WHR =  Waist-to-Hip ratio; BMI = Body mass index; WC = Waist circumference; BP = Blood pressure; FPG =  Fasting plasma glucose; DD =  duration of diabetes.

Shown in table 2 is the prevalence of distortion, anthropometric and clinical parameters. Thirteen percent of the participants were not hyperglycaemic and 45.0% (n = 90) were hypertensive. More than half (58.0%, n = 116) of the participants had abdominal obesity, which was significantly (p<0.001) higher in women than in men. Whereas 32.0% were found to have general *overweight/obesity (measured by BMI)*, only 13.0% (n = 26) perceived themselves to be overweight/obese. Fifty-eight percent (58.0%) of the study participants had a distorted weight perception in which 77.6% (n = 90) underestimated their weight status. Less than 30% of the participants ever tried to lose weight.

**Table 2:** Demographic and prevalence of distortion, anthropometric and clinical parameters.

Variable	Total (n = 200)	Men (n = 46)	Women (n = 154)	P value
Hyperglycaemic	154(77.0%)	34(73.9%)	120(77.9%)	0.556
Hypertensive	90(45.0%)	26(56.5%)	64(41.6%)	0.091
Abdominally obese	116(58.0%)	6(13.0%)	110(71.4%)	<0.001
Self-perceived Underweight	62(31.0%)	12(26.1%)	50(32.5%)	0.471
Self-perceived Normal weight	122(61.0%)	28(60.9%)	94(61.0%)	1.000
Self-perceived Overweight	26(13.0%)	10(21.7%)	16(10.4%)	0.077
Overweight/obese (BMI)	64(32.0%)	16(34.8%)	48(31.2%)	0.719
Distorted weight perception	116(58.0%)	30(65.2%)	86(55.8%)	0.308
Underestimation of weight status	90/116 (77.6%)	20(43.5%)	70(45.5%)	0.867
Ever tried to lose weight	54(27.0%)	12(26.1%)	42(27.3%)	1.000
Ever tried to gain weight	22(11.0%)	6(13.0%)	16(10.4%)	0.770

### Distorted self-perceived weight status

**Figure 1:**
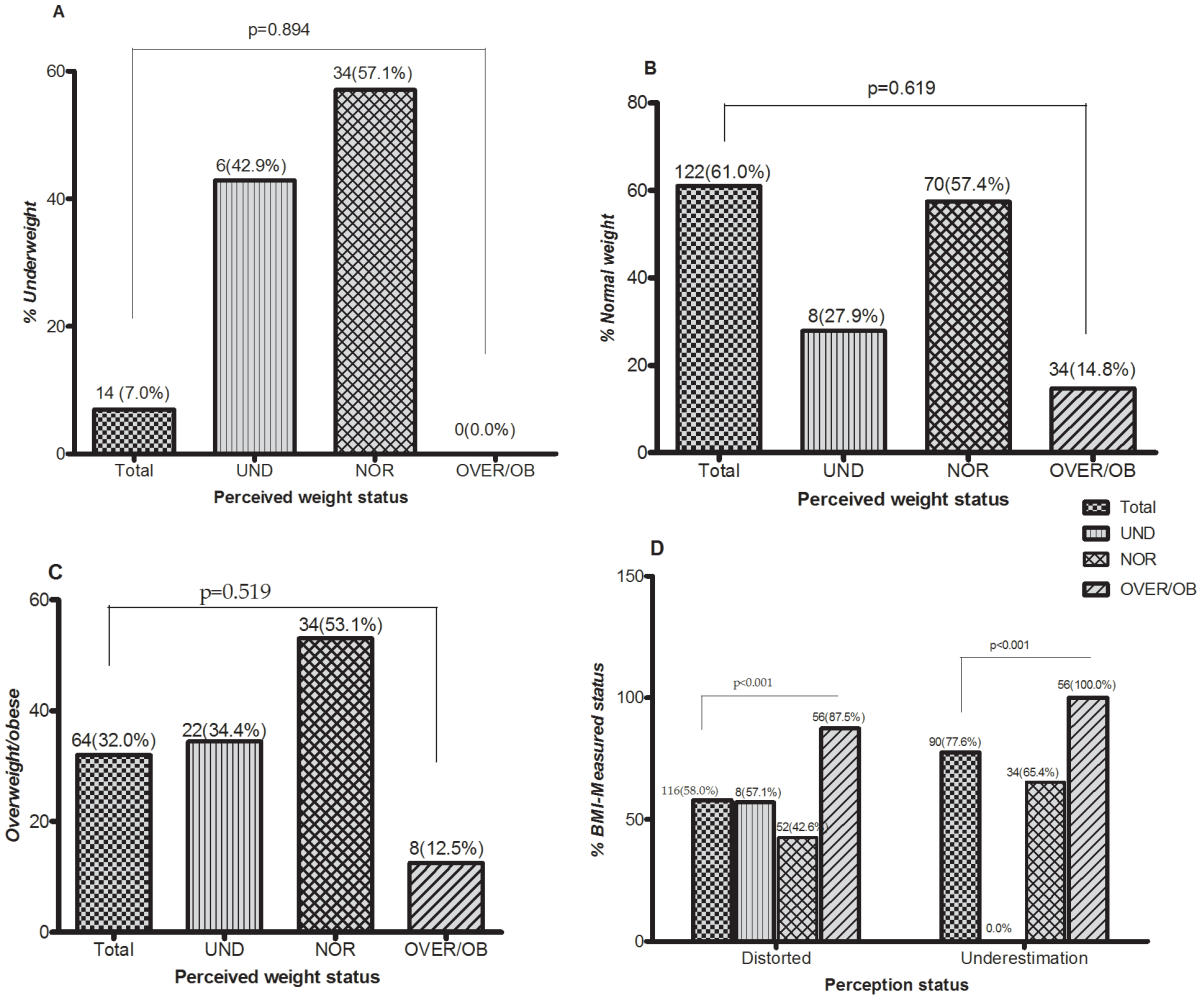
*BMI-measured weight status* compared to self-perceived weight status. Comparison between *BMI-measured weight status* with perception of underweight (A); perception of normal weight (B); perception of overweight/obese (C) and perception status (D). UND =  Underweight, NOR = Normal weight, OVER/OB =  Overweight/obesity. Data was presented as proportion and analyzed using chi-square for trend.

*BMI-measured* weight *status* was compared with Self-perceived weight status and presented in figure 1. Over 40% of the participants self-perceived themselves as underweight when in fact only 7.0% (n = 14) of them were *underweight* (Figure 1A). Poor agreement was observed between self-perceived weight status and *BMI-measured weight* (Kappa  = 0.025, SE = 0.046, CI = −0.065–0.116, p = 0.571). In figure 1C, only 12.5% (n = 8) of the participants perceived themselves as overweight/obese when in fact 32.0% (n = 64) of them were *overweight/obese*. As shown in figure 1D, distorted self-perceived weight status increased significantly (p<0.001) with an increase in *BMI-measured* weight status. As *BMI-measured* weight status increased, the prevalence of underestimation of weight status increased significantly (p<0.001) (shown in figure 1D).

### Factors associated with underestimation of weight status

Using univariate analysis, we identified factors associated with underestimation of weight status among the participants and shown in table 3. Participants aged >40 years had a 4.2 (95% CI = 1.37–12.93, p = 0.011) crude odds ratio of underestimating their weight status. Other factors that were found to be associated with underestimation of weight status were being not married; had never tried to lose weight overweight*/obese* and hyperglycaemic.

**Table 3:** Univariate analysis of factors associated to underestimation of weight status.

Variable	Response	n/N*	ROU	COR (95% CI)	P value
**Gender**	Male	20/46	43.5%	1	1
	Female	70/154	45.5%	1.1 (0.56–2.10)	0.867
**Age (years)**	≤40	4/22	18.2%	1	1
	Over 40	86/178	48.3%	4.2 (1.37–12.93)	0.011
**Marital status**	Married	60/152	39.5%	1	1
	Not married	30/48	62.5%	2.6 (1.31–4.99)	0.007
**Educational status**	High	38/68	55.9%	1.5 (0.84–2.74)	0.181
	Low	60/132	45.5%	1	1
**Tried to lose weight**	Yes	16/54	29.6%	1	1
	No	74/146	50.7%	2.4 (1.25–4.76)	0.010
**Tried to gain weight**	Yes	12/22	54.5%	1.5 (0.63–3.74)	0.371
	No	78/178	43.8%	1	1
**Hypertensive**	Yes	36/90	40.0%	1	1
	No	54/110	49.1%	1.4 (0.82–2.54)	0.253
**Hyperglycaemic**	Yes	82/154	53.2%	5.4 (2.37–12.35)	<0.001
	No	8/46	17.4%	1	1
**Overweight/obese (BMI)**	Yes	56/64	87.5%	21.0 (9.10–48.47)	<0.001
	No	34/136	25.0%	1	1
**Abdominal obesity**	Yes	58/116	50.0%	1.6 (0.92–2.88)	0.114
	No	32/84	38.1%	1	1

*Number of subjects with underestimation of weight status in each category; ROU =  Rate of underestimation; COR =  Crudes odds ratio.

Table 4 presents the results of a multiple logistic regression analysis of factors affecting underestimation of weight status among the participants. After controlling for several factors, the adjusted odds of underestimating weight were being not married (AOR = 3.7, 95% CI = 1.50–9.17, p = 0.005); never tried to lose weight (AOR = 6.9 95% CI = 2.35–19.97, p<0.001) and being *overweight/obese* (AOR = 22.9, 95% CI = 8.30–63.07, p<0.001). Age and hyperglycaemic status were not significantly associated to underestimation of weight status.

**Table 4:** Multiple logistic regression analysis of factors associated to underestimation of weight status.

Variable	Response	B	AOR (95% CI)	P value
Age (years)	≤40	1	1	1
	Over 40	0.63	1.9 (0.46–7.66)	0.384
Marital status	Married	1	1	1
	Not married	1.31	3.7 (1.50–9.17)	0.005
Ever tried to lose weight	Yes	1	1	1
	No	1.93	6.9 (2.35–19.97)	<0.001
Hyperglycaemic	Yes	1	1	1
	No	−0.81	0.44 (0.17–1.18)	0.103
Overweight/obese (BMI)	Yes	3.13	22.9 (8.30–63.07)	<0.001
	No	1	1	1

Cox and Snell R^2^ = 0.39; Nagelkerke R^2^ = 0.53; AOR = Adjusted odds ratio.

## Discussion

### Main results

High prevalence of general and abdominal obesity was observed in this diabetes population in Tamale, Ghana. Distorted self-perceived weight status was prevalent with over 70% of them underestimating their weight status. We observed that as *BMI-measured* weight status increased, distorted and underestimation of self-perceived weight status increased significantly. This resulted in the *overweight/obese* participants being several folds at risk of underestimating their weight status. This is of grave concern since correct perception of one's weight status is strongly associated with positive efforts to lose weight and maintain a healthy lifestyle [29].

### Comparison with other studies

The prevalence of general *overweight/obesity (BMI-measured)* was found to be 32.0%. Taking cognizance of variations in the range of definitions of overweight/obesity, the prevalence of *overweight/obesity* in this study can be said to be among the lowest rates reported in the literature among diabetes patients. A study conducted among diabetes patients in an urban area showed that 82.8% of men and 79.6% of women were either *overweight or obese* [11]. Recently, a study in Kumasi, Ghana among diabetes patients found the prevalence of *overweight/obesity* to be 72.0% [2]. In the UK, Dauosi et al. [8], reported that 86% of the study participants with type 2 diabetes were *overweight or obese*.

Another important finding of our study was that the prevalence of abdominal obesity measured by Waist circumference (WC) (58.0%) was found to be higher than general *overweight/obesity (BMI-measured)*. This is of grave concern since WC has been shown to be associated with risk of obesity-related morbidity and mortality [30]. Abdominal fat deposition measured by WC has been shown to be a better indicator of obesity in relation to metabolic syndrome, type 2 diabetes, and cardiovascular diseases than BMI [31], [32], [33]. Also, WC has been reported to increase at a faster rate than BMI, and the adverse health consequences of obesity may be underestimated by measuring only BMI in patients with diabetes [34]. Measurement of WC is a simple and inexpensive method, which should be done regularly in the treatment and management of diabetes [35]. In fact the US National Institutes of Health recommended that WC be measured to screen for health risk, especially among those with a BMI more than 25.0 kg/m^2^ [36].

Hypertension is a common comorbid condition in diabetes mellitus type 2 (DM 2) and vice versa [37]. Hypertension and diabetes mellitus coexist in approximately 40% to 60% in patients with DM 2 [37], [38], [39]. It is therefore not surprising that over 40% of the participants in our study were hypertensive. This has been observed in several studies conducted in Ghana [2], [40], [41] and several other countries [42], [43], [44].

A high prevalence of distorted self-perceived weight status was found, with over 70% of the participants underestimating their weight status. Consistent with our findings, a study in adult inhabitants of Cracow, Poland reported misperception of weight status in people with diabetes and coronary heart disease [45]. Another study in a multi-ethnic urban cohort in Dallas, the United states [46] reported a poor weight status perception in people with comorbid conditions.

Distortion and underestimation of self-perceived weight status was found to increase significantly with an increase in *BMI-measured* weight status. From our univariate and multiple logistic models, *overweight/obese* participants were several folds at risk of underestimating their weight status compared to their *normal weight* counterparts. A study among 575 overweight adults with type 2 diabetes found misperception of weight to be rare (<10%) among patients with BMI>30, but more common (47%) among patients with BMI<30, with most of them underestimating the weight that would be healthiest for their height [47]. McTigue et al. [11], reported contrary results in a study among patients with diabetes in which most overweight (95%) or obese (99%) respondents correctly self-perceived their weight status as overweight. The differences may be related to race and geographical locations.

Another important finding of this study was that married participants were less likely to underestimate their weight status as compared to their unmarried counterparts. In our univariate and multiple logistic regression models, we found that unmarried (which included singles, divorced and widows) participants had a higher odds of underestimating their weight status and less likely to perceive themselves as overweight/obese compared to married participants. Consistent with our findings Burke and Colleagues [48] in a study to identify differences in the self-perception of weight status in the United States between two National Health and Nutrition Examination Survey (NHANES) periods (1988–1994 and 1999–2004) revealed that married people were more likely to overestimate their weight status than never-married people.

In our study, participants aged 40 years and over were more likely to underestimate their weight status, which is consistent with several studies [45], [49], [50]. Even though, the association between age and underestimation of weight status was not significant in our multiple logistic regression model, the high proportion of weight status underestimation among the older group could be due to the usual acceptance of overweight and obesity as an age related occurrence [51]. This is a cause of concern especially among diabetes patients in which the elevated BMIs may be falsely associated to age. As a result patients may not readily adopt recommendations to lose their weight. This has been established in this study such that participants who have never tried to lose weight were more likely to underestimate their weight status.

### Strengths and limitations

Our study is not without limitations. Firstly, our study was a cross-sectional one which is unable to establish causality. Secondly, this study was conducted in an urban setting which cannot be generalized to the entire Ghanaian population. However, it provides a basis upon which future extensive studies can be conducted. Thirdly, our study did not measure body size dissatisfaction and body image perception which are important factors of weight perception and weight loss attitudes. Finally, fasting plasma glucose and blood pressure values were obtained secondarily from the personal health files of the diabetes patients. Although, all care was taken to record the values to minimize errors, misreporting might have occurred. Also, distortion of weight status was measured by a single question which has the potential of misclassification. However, this form of assessment of distortion of weight status has been validated in several studies [14], [15], [49].

### Recommendations and conclusions

Given the importance of perception of body weight in managing diabetes, our findings elicit a cause for concern. Weight misperception has been shown to be related to unhealthy life styles [49] which may contribute to the development and progression of chronic diseases such as DM 2. This has been shown in this study in which underestimation of weight status was associated to hyperglycaemia, indicating a progression of the condition. Also participants who had never tried to lose weight were more likely to underestimate their weight status, indicating the practice of unhealthy lifestyles which, probably was influenced by their self-perceived underestimation of weight status. Distorted self-perceived weight status could influence the perceived relevance of health recommendations and the efficacy of weight management interventions [52]. This would make prevention and treatment efforts more challenging. Patients are unlikely to adopt attitudes to prevent or treat overweight or obesity if they do not perceive their weight status as problematic or if they do not consider the diabetes as a cause of it [15], [53], [54], [55], [56]. Health care providers should make efforts in providing patients with information about weight guidelines.

Distorted self-perceived weight status is attributable to knowledge, beliefs and social circumstances [15], [24], [49], [55]. These should be taken into account when designing preventive and treatment interventions for DM 2 patients. It is therefore imperative that DM 2 patients should be able to correctly perceive their weight as well as having knowledge of health risk associated with obesity. Also further extensive qualitative studies should be conducted to identify social and psychological factors that are affecting underestimation of weight status in DM 2 patients.
